# Ultra-high-performance liquid chromatography-atmospheric pressure ionization-tandem mass spectrometry method for the migration studies of primary aromatic amines from food contact materials

**DOI:** 10.1007/s00216-022-03946-3

**Published:** 2022-03-02

**Authors:** Ane Arrizabalaga-Larrañaga, Pedro de Juan-de Juan, Claudia Bressan, Mercedes Vázquez-Espinosa, Ana V. González-de-Peredo, F. Javier Santos, Encarnación Moyano

**Affiliations:** 1 Department of Chemical Engineering and Analytical Chemistry, University of Barcelona, Av. Diagonal 645, 08028 Barcelona, Spain; 2 Department of Analytical Chemistry, Faculty of Sciences, Agrifood Campus of International Excellence (ceiA3), University of CádizIVAGROPuerto Real, 11510 Cádiz, Spain

**Keywords:** Primary aromatic amines, Atmospheric pressure ionization, Liquid chromatography-tandem mass spectrometry, Atmospheric pressure chemical ionization, Pentafluorophenylpropyl column, Food contact materials

## Abstract

This work describes the development of an ultra-high-performance liquid chromatography-tandem mass spectrometry (UHPLC-MS/MS) method for the determination of 23 primary aromatic amines (PAAs) that can potentially migrate from food contact materials. The chromatographic separation was performed in a pentafluorophenylpropyl (PFPP) column achieving the separation of all PAAs in less than 6.5 min using water to acetonitrile (0.1% acetic acid in both solvents) as mobile phase and a gradient elution. The feasibility of atmospheric pressure chemical ionization (APCI) and atmospheric pressure photoionization (APPI) was evaluated as alternative to electrospray ionization (ESI) for the analysis of PAAs. Results showed that for most of the compounds, better responses were obtained with APCI, which shows the advantage of being less susceptible to matrix effects. Tandem mass spectrometry (MS/MS) fragmentation studies of [M + H]^+^ allowed for the selection of the two most characteristic and abundant product ions of the 23 PAAs which led to the development of a selective and sensitive UHPLC-APCI-MS/MS method with limits of detection ranging from 0.2 to 2 μg kg^−1^. Moreover, intra-day and inter-day precisions of the method in terms of relative standard deviation (RSD%) were lower than 10% and 15%, while trueness as relative error was <15% for most of the compounds. The UHPLC-APCI-MS/MS method was applied to the analysis of twenty black Nylon kitchenware samples that were submitted to migration tests using food simulant B (3% acetic acid, *w/v*), and the presence of PAAs were detected in eighteen samples at concentrations above the legislated limit (2 μg kg^−1^ of food or food simulants).

**Graphical abstract:**

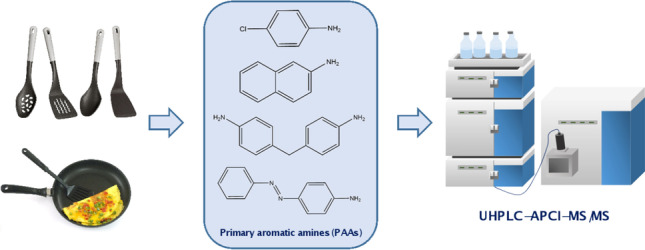

**Supplementary Information:**

The online version contains supplementary material available at 10.1007/s00216-022-03946-3.

## Introduction

In recent years, the concern about the possible migration of components from food contact material (FCM) into food has increased considerably in the food safety field. The European Union law, within the framework of the Regulation 1935/2004/EC [1], established the good manufacturing practices of materials and objects intended to come into contact with food. Nevertheless, in recent years, a large number of notifications have been issued by RASFF (Rapid Alert System for Food and Feed) concerning the migration of primary aromatic amines (PAAs) from FCM. According to the World Health Organization and the International Agency for Research on Cancer (IARC) [2], some PAAs have shown carcinogenic and genotoxic effects in humans and they have been classified into different toxicological groups. Thereby, the European Union, within the framework of the Regulation 1245/2020/EC [3], has stated specific migration limit for the release of PAAs at 2 μg of individual PAAs per kilogram of food or food simulants. Some of the main sources of PAAs in food are due to the use of multilayer food packaging materials since they use isocyanate compounds as polyurethane adhesives [4], as well as in polyamide kitchenware [5, 6]. For instance, various studies have reported the migration of PAAs into food from multilayer materials [7, 8]: black Nylon kitchen utensils [9, 10].

Several analytical methodologies have been reported in the literature for the determination of some PAAs in food contact materials. Concerning the separation techniques used, gas chromatography (GC) [11, 12] is the less frequently used since it requires a derivatization step. Capillary electrophoresis (CE) [13, 14] has also been proposed although limits of detections (LODs) are not low enough to determine PAAs in migration tests of food simulants or food samples without previous preconcentration steps. Thereby, liquid chromatography (LC) is the technique most commonly used for the chromatographic separation of PAAs [15–18]. Generally, these methodologies use both reversed-phase C18 columns specially designed for the separation of basic compounds with neutral mobile phases to avoid the protonation of PAAs and enhance their chromatographic retention [7, 19–22]. Besides, to improve the retention of PAAs in reversed-phase columns, ionic-pair reagents such as pentafluoropropionic acid (PFPA) have also been proposed [7, 9]. So far, hydrophilic interaction chromatography (HILIC) [24] and phenyl-hexyl columns [10] have also been applied for the separation of PAAs, but few PAAs (5–8 compounds) were included in these studies. Besides, as far as we know, a pentafluorophenylpropyl (PFPP) column, which combines both hydrophobic and hydrophilic interactions to retain analytes, has not yet been evaluated for the chromatographic separation of PAAs in migration studies from FCMs. Regarding the employed detection system, liquid chromatography coupled to tandem mass spectrometry (UHPLC–MS/MS) in multiple reaction monitoring (MRM) acquisition mode is the technique of choice for the determination of PAAs at low concentration levels in food simulants and food samples. Since PAAs are easily ionized in a liquid phase at acidic pH values, electrospray (ESI) has been the most commonly used ionization source. To the best of our knowledge, no data has been reported evaluating the performance of other atmospheric pressure ionization (API) sources, such as atmospheric pressure chemical ionization (APCI) and atmospheric pressure photoionization (APPI), for the ionization of a large number of PAAs and it would be very interesting to have information about its capabilities and performance in the ionization of these compounds.

The present work aimed to develop a new ultra-high-performance liquid chromatography coupled to tandem mass spectrometry (UHPLC–MS/MS) method for the determination of PAAs that can migrate from food contact materials. For this purpose, the chromatographic behavior of PAAs with different stationary phases and avoiding the use of ion-pair reagents was evaluated. Besides, the ionization performance of PAAs with the three API sources (ESI, APCI, and APPI) and their tandem mass spectrometry fragmentation were studied to characterize and assign the relevant product ions and select those for quantitation and confirmation purposes. Finally, the developed UHPLC-MS/MS method was applied to the analysis of 23 PAAs in migration tests of kitchenware materials.

## Experimental

### Chemicals and materials

Standards and chemicals used in this work were of analytical grade. Twenty-three aromatic primary amines were included in this study and their acronym, CAS number, chemical formula, pKa, and log *P* values, as well as the chemical structure, are given in Table 1. Pure standards of 2,4-toluenediamine (2,4-TDA); 2,6-toluenediamine (2,6-TDA); 2,4,5-trimethylaniline (2,4,5-TRA); 2-methoxy-5-methylaniline (2-M-5-MA); 4-methoxy-*m*-phenylenediamine (4-M-*m*-PDA); *p*-cloroaniline (*p*-CA); 2-naphthylamine (2-NAP); 1,5-diaminonaphthalene (1,5-DAN); benzidine (BNZ); *p*-aminoazobenzene (*p*-AAB); 4,4′-methylenedianiline (4,4′-MDA); 4,4′-oxydianiline (4,4′-ODA); 3,3′-dimethylbenzidine (3,3′-DMB); 4,4′-thiodianiline (4,4′-thioDA); 3,3′-dichlorobenzidine (3,3′-DCB); and 4.4′-methylene-bis-(2-chloroaniline) (4,4′-M-2-CA) were purchased from Sigma-Aldrich (Steinheim, Germany), while *o*-toluidine (*o*-T), *o*-anisidine (*o*-ASD), *p*-chloro-*o*-methylaniline (*p*–C-*o*-MA), *p*-aminobiphenyl (*p*-ABP), *o*-aminoazotoluene (*o*-AAT), and *o*-dianisidine (*o*-diASD) were supplied by Alfa-Aesar (Haverhill, MA, USA) and aniline (ANL) from Panreac (Barcelona, Spain). Acetone for pesticide residue analysis (LiChrosolv®, ≥ 99.8%) was obtained from Honeywell (Charlotte, NC, USA), while anisole anhydrous (≥ 99.7%), tetrahydrofuran (THF) (≥ 99.7%), toluene, and chlorobenzene for HPLC (≥ 99%); ammonium formate (≥ 98%); formic acid (≥ 98%); ammonium acetate (≥ 98%); acetic acid (≥ 97%); and water, methanol, and acetonitrile of LC–MS grade were purchased from Sigma-Aldrich (Steinheim, Germany). Solvents used in the mobile phase were filtered through 0.22-μm pore size Nylon membrane filters (Whatman, Clifton, NJ, USA) before their use.

**Table 1:**
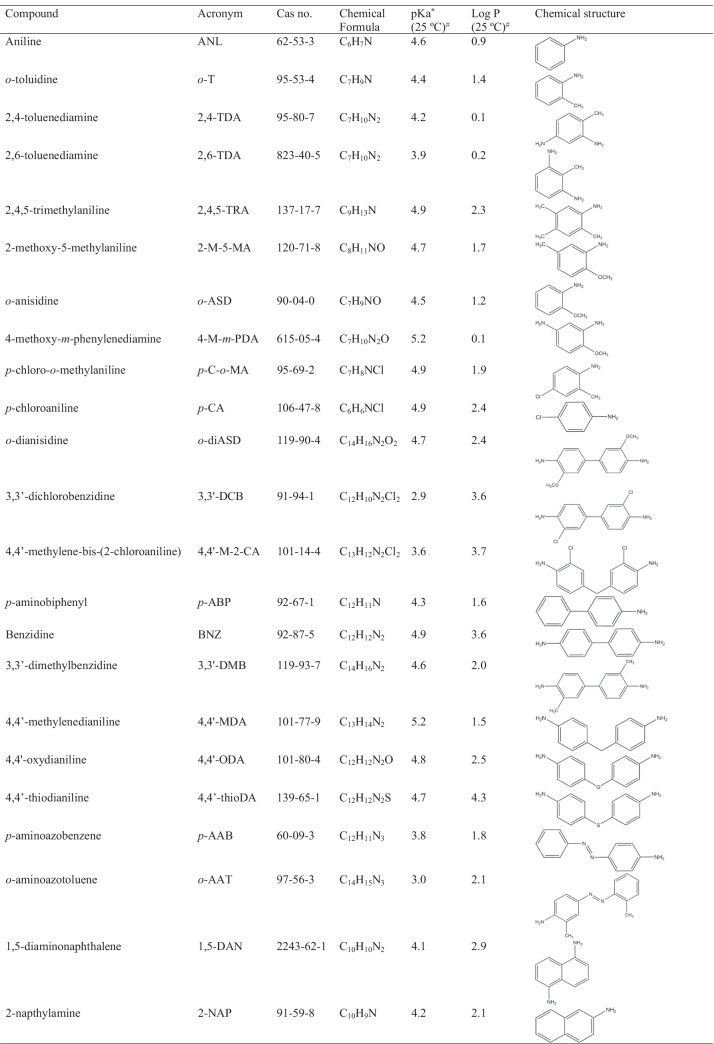
Amines, their abbreviated name, CAS number, chemical formula, pKa and log *P* values, and chemical structure

^*^pKa values of conjugated acid

^#^Values estimated by the software percepta (ACD/Labs)

Stock standard solutions of PAAs (1000 mg kg^−1^) were individually prepared by weight in methanol and stored at 4 °C, while an intermediate standard mixture containing all the target compounds (20 mg kg^−1^) was prepared weekly by dilution in food simulant B (3% acetic acid, *w/v*). For quantitation, calibration solutions were prepared from the intermediate standard solution at concentrations ranging from 0.5 to 10,000 μg kg^−1^ in 3% acetic acid (*w/v*).

### Instrumentation and UHPLC-MS/MS conditions

The chromatographic separation of primary aromatic amines was carried out on an Accela UHPLC system (Thermo Fisher Scientific, San Jose, CA, USA) equipped with a quaternary pump, an autosampler, and a column oven. Four UHPLC columns (superficially porous particles) were evaluated: (1) Accucore C_18_ (100 × 2.1 mm, 2.6 μm), (2) Accucore Phenyl-Hexyl (150 × 2.1 mm, 2.6 μm), and (3) Accucore HILIC (100 × 2.1 mm, 2.6 μm) from Thermo Fisher Scientific, and (4) Ascentis Express F5 (pentafluorophenylpropyl, PFPP) (100 mm × 2.1 mm, 2.7 μm) provided by Supelco (Belllefonte, PA, USA). The mobile phase used for the chromatographic separation under the optimal conditions for the Ascentis Express F5 column was 0.1% acetic acid aqueous solution (solvent A) and 0.1% acetic in acetonitrile (solvent B). The gradient elution program was as follows: 0–1 min, isocratic conditions at 90% solvent A; 1–8 min, linear gradient elution from 90 to 10% A; 8–10 min, isocratic conditions 10% solvent A. Mobile phase flow-rate was 500 μL min^−1^, injection volume (in full loop mode) was 10 μL, and column oven temperature was held at 25 °C during the chromatographic run.

The UHPLC system was coupled to a TSQ Quantum Ultra AM (Thermo Fisher Scientific) mass spectrometer equipped with a triple quadrupole mass analyzer. Three API sources could be swappable in the TSQ mass spectrometer, ESI, APCI, and APPI (Thermo Fisher Scientific). The nitrogen (99.95%) used for the three API sources was purchased from Linde (Barcelona, Spain). The ionization source working conditions were optimized by infusing 10 μL min^−1^ with a built-in syringe pump. The individual standard solutions (300 mg kg^−1^) were infused through the mobile phase stream (300 μL min^−1^) using a Valco zero dead volume T-piece. The H-ESI, APCI, and APPI optimal working conditions were as follows: sheath gas and auxiliary gas pressures were 70 and 60 a.u. (arbitrary units), respectively, and vaporizer and ion transfer tube temperatures were held at 400 °C and 300 °C, respectively. Besides, in H-ESI, the spray voltage was + 4 kV, and the corona discharge current in APCI was + 5 μA, whereas the krypton lamp emitted 10.6 eV photons in APPI. The tube lens offset voltage was optimized for each compound and the optimal values ranged from 73 to 115 V. Concerning APPI, the selected solvent as a dopant was chlorobenzene, which was post-column added to the mobile phase stream at 10 μL min^−1^ using a T-piece (Valco).

Data were acquired in both full scan and product ion scan modes for fragmentation studies, whereas multiple reaction monitoring mode was used for quantitation purposes (Table S1). Both quadrupoles (Q1 and Q3) operated always at a resolution of 0.7 m*/z* full width half maximum (FWHM) and two transitions were monitored for each compound using 50-ms dwell time. High-purity argon (Ar_1_) (< 99.999%) supplied by Air Liquide (Madrid, Spain) was used as collision-induced dissociation gas (CID gas) at a pressure of 1.5 mTorr. The Xcalibur software *v*4.0 (Thermo Fisher Scientific) was used to control the UHPLC-API-MS/MS system and to acquire and process the mass spectrometry data.

### Samples and sample preparation

A total of twenty black Nylon kitchenware utensils (spatula (2), spoons (6), slotted spatula (3), slotted spoons (4), slotted turner (3), potato masher (1), and pasta server (1)) were purchased from different local markets in Barcelona (Spain) and analyzed in this work. The migration tests of PAAs performed on the kitchenware samples were carried out following the European Commission technical guide^6^ and the European Standard EN 13,130–1:2004 [25]. Since the surface-to-volume ratio in the total immersion tests should be 600 cm^2^ of food contact material for 1000 mL of food simulant^18^, the size of the sample surface to be tested was scaled to use a smaller volume of food simulant. Thereby, the surface of plastic laminate samples was cut into small pieces of 1 cm^2^ (2 cm^2^ total contact surface) and it was immersed in 3.3 mL of food simulant B (3% acetic acid, *w/v*) weighed in a 40-mL glass vial. The closed vial (to prevent evaporation) was warmed up at 100 °C for 2 h in an oven. After cooling down, the sample extract was filtered through 0.22-μm pore size Nylon membrane filters (Whatman, Clifton, NJ, USA) and transferred into an injection vial. This procedure was carried out three consecutive times with the same sample using fresh food simulant (1st, 2nd, and 3rd migration test) following the Regulation 1245/2020/EC [3] which requires three successive migration tests for articles intended for repeated use and consider as final test result that obtained in the third test. Each kitchenware was analyzed in triplicate and the calibration solutions were prepared in food simulant B to quantify the PAAs released from samples.

### Quality control and method validation

To guarantee the quality of the data, specific tests to check both chromatographic separations and the sensitivity of the UHPLC-MS/MS system were carried out using standards and quality controls prepared in food simulant. Limits of detection (LOD) was estimated based on a signal-to-noise (S/N) ratio of 3. Moreover, linearity was studied within the working concentration range (0.5 to 10,000 μg kg^−1^), and both intra-day and inter-day precisions and trueness of the method were also estimated using spiked blank food simulant at three concentration levels: 2, 10, and 20 μg kg^−1^.

## Results and discussion

### Liquid chromatography

To achieve the best UHPLC-API-MS performance, the chromatographic separation conditions should be compatible with the atmospheric pressure ionization technique. These targeted compounds have more than one amino group in the chemical structure, leading to more than 1 pKa value. Since the first pKa value of the conjugated acid ranges from 2.9 to 5.2 and the log *P* value of neutral PAAs ranges from 0.1 to 2.3, they show a high hydrophilic character (Table 1). These characteristics indicate that mobile phases with pH values lower than 3.0 should be used to favor the protonation of all PAAs in the liquid phase. When injecting a standard mixture containing the 23 PAAs in a reversed-phase C_18_ column, only the use of neutral mobile phase (methanol to water) permitted to achieve a satisfactory separation, but the ESI signal was very poor for all PAAs. The acidification of the mobile phase increased the response in ESI but reduced the chromatographic retention of PAAs, which made most of them to elute close to the elution front. Thereby, a post-column addition of 0.1% acetic acid (AcOH) was carried out as an alternative strategy for improving the ionization of PAAs in ESI. The aforementioned approach allowed the reduction of the pH of the mobile phase after achieving the satisfactory chromatographic separation of PAAs using a neutral mobile phase (Fig. S1A). However, since a post-column addition would add an extra difficulty to the routine analysis in a control laboratory, in the present work, we explored other alternative strategies to retain PAAs without sacrificing ionization efficiency.

Some authors have proposed the addition of ion-pair reagents to the mobile phase to form and retain ion-pair species (more hydrophobic) with PAAs [7, 19]. Nevertheless, the use of mobile phases with ion-pair reagents are not recommended in LC–MS, since they could produce accumulation of nonvolatile components causing contamination of the LC–MS system and ion suppression problems, so this strategy was discarded. Thereby, in the present work, the applicability of other stationary phases that offered alternative retention mechanisms was explored. UHPLC analytical columns with phenyl-hexyl, bare silica (HILIC), and pentafluorophenylpropyl as stationary phases, as alternative to the classical reversed-phase C_18_, were evaluated for the LC separation of 23 PAAs using acidic mobile phases. The phenyl-hexyl column can undergo hydrophobic interactions through the hexyl chain moiety and π-π interactions by the phenyl group, which could be useful to retain and separate the PAAs. The standard mixture of PAA was injected in the phenyl-hexyl column using a methanol to water (0.1% formic acid, *v/v*) gradient elution program. Under these chromatographic conditions, PAAs were strongly retained in the phenyl-hexyl column than in the C_18_ column providing less peak tailing (Fig. S1B). However, the 2,4-TDA showed more peak tailing in the chromatogram corresponding to the individual standard solution than in that obtained when injecting the standard mixture. This phenomenon might be related to the partial coelution of ANL, which produced ion suppression in the 2,4-TDA peak and distorted the shape of the peak. Although the baseline separation of these compounds would prevent problems in their quantitative analysis, it was not possible to achieve it under any chromatographic conditions tested.

HILIC column was also tested since the mixed-mode retention mechanism based on the partition of analytes between the organic-rich mobile phase, the partially immobilized water-enriched layer, and the electrostatic interactions and hydrogen bonds were expected to help in the separation of PAAs using acidic mobile phases [26]. The standard mixture of PAAs was injected in the HILIC column using an isocratic elution and a mobile phase with low eluotropic strength such as acetonitrile to water (90:10, *v/v*) and acetonitrile: 0.1% aqueous formic acid (90:10, *v/v*). The retention of PAAs was in inverse elution order than that obtained with the C_18_ column (Fig. S1C). However, the HILIC column provided broad tailing peaks for most of target amines, probably owing to the strong interaction of cationic species (protonated amines) with the silanol groups on the silica surface.

**Fig. 1:**
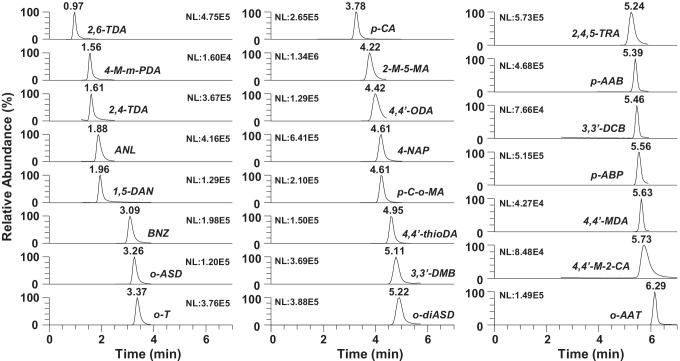
UHPLC-MS/MS chromatogram obtained from a standard mixture of target PAAs at a concentration of 1 mg kg^−1^

With the last column tested, a pentafluorophenylpropyl column, the perfluorinated phenyl group and the short alkyl chain (propyl) provided a selectivity based on the enhanced dipole, π-π, charge transfer, and ion-exchange interactions [27]. The standard mixture of 23 PAAs was injected in isocratic elution mode using different percentages of organic modifier (10–90%) in mobile phases based on mixtures of methanol to water (0.1% formic acid) and acetonitrile to water (0.1% formic acid). The plot of retention time of PAAs versus the percentage of organic modifier showed a U-shape (Fig. S2), which indicated that mobile phases with high water content favored the dispersive (hydrophobic) interactions allowing a stronger retention of PAAs. In contrast, mobile phases with high organic modifier content (above 60%) favored the ion-exchange interactions on the silica surface, which controlled and favored the retention of protonated PAAs. The best separation for most critical PAA pairs (2,6-TDA/2,4-TDA and 1,5-DAN/ANL) were obtained when using acetonitrile-based mobile phases. Moreover, acetonitrile to water mobile phases at pH below 5.2 (the highest pKa value of PAAs) adjusted using acidic aqueous solutions that contained 0.1% formic acid (pH 2.6), 0.1% acetic acid (pH 3.2), or acidic buffer solutions such as 50 mM formic acid/ammonium formate (pH 3.7) and acetic acid/ammonium acetate (pH 4.6) were also evaluated. A linear gradient elution mode from 10 to 70% of organic modifier was applied to obtain the best separation of PAAs avoiding the retention mechanisms through ion-exchange interactions and also the distortion of the chromatographic peaks, generally observed when working with over 70% of acetonitrile. Moreover, the highest chromatographic efficiency and resolution were obtained when using mobile phases with lower pH values (pH 2.6 with 0.1% formic acid and pH 3.2 with 0.1% acetic acid). However, the lower ionic strength of the mobile phase containing 0.1% acetic acid allowed a stronger retention of PAAs, as occurred in ion-exchange separations, but it also had a positive effect on the ionization efficiency as it helped in the solvent evaporation processes. Therefore, 0.1% acetic acid was added to both water and acetonitrile to control the pH and the ionic strength of the mobile phase through the chromatographic separation. Besides, ion suppression and ion enhancement of unresolved compounds (Rs < 1.5) was checked and the results showed that differences observed between the obtained responses from individual standard solutions and those observed when analyzing the standard mixture of PAAs were lower than 7% in all API sources tested, which indicated that ion suppression/enhancement could be considered negligible for these compounds. After comparing the chromatographic behavior of PAAs in the different UHPLC columns tested, the PFPP column with acidic mobile phase was proposed for subsequent studies. This chromatographic system provided the best chromatographic separation of 23 PAAs in less than 6.5 min (Fig. 1) and favored the optimal ionization of PAAs with API sources without the need to use any ion-pair reagents and post-column additions.

### Liquid chromatography-mass spectrometry

**Fig. 2:**
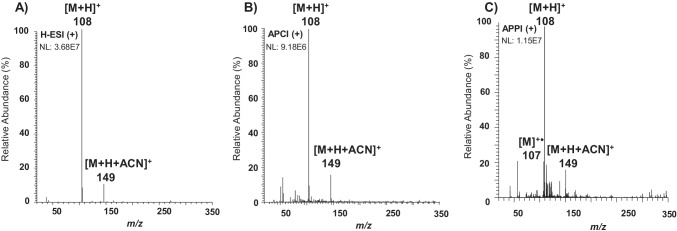
Full-scan mass spectra of *o*-T using **A** H-ESI, **B** APCI, and **C** chlorobenzene-assisted APPI in positive-ion mode

The atmospheric pressure ionization behavior of targeted PAAs was studied using H-ESI, APCI, and APPI sources. Although the base peak was for all the API sources, the protonated molecule [M + H]^+^, most of PAAs also showed a tendency to generate adduct ions with acetonitrile [M + H + ACN]^+^. When the vaporizer temperature was raised to 400 °C, there was a significant improvement in both desolvation of target compounds and the signal of the protonated molecule [M + H]^+^, although adduct ions could not be fully avoided (Fig. S3). Table 2 shows the ions generated by H-ESI, APCI, and APPI in positive-ion mode under optimal conditions (see “Instrumentation and UHPLC-MS/MS conditions”). As an example, Fig. 2 depicts the full-scan mass spectra of *o*-T using the H-ESI (Fig. 2A), APCI (Fig. 2B), and APPI (Fig. 2C). In APPI, PAAs yielded the ion [M + H]^+^ as the base peak of mass spectra due to the high proton affinity of the amino group, as well as the acetonitrile adduct ions. Additionally, the molecular ion [M]^+•^ was also present in the APPI mass spectra of PAAs indicating that these compounds could be photoionized without the use of dopants (D), probably due to their low ionization potential (*ca.* 7.5 eV). Despite the possibility of direct ionization, several dopants (acetone, toluene, THF, chlorobenzene, and anisole) were tested to improve the ionization efficiency of PAAs. Among the evaluated dopants, acetone, toluene, and THF provided similar mass spectra in APPI than those previously observed in H-ESI and APCI. It should be noted that acetone and THF showed self-protonation in the gas phase leading to the protonated dopant [D + H]^+^ and this process would prevent the possibility of ionizing PAAs through a charge exchange mechanism [28]. In the case of toluene, the protonation of PAAs might occur through proton transfer reactions where both the dopant molecular ion and the reactive species generated from the mobile phase are involved. Otherwise, chlorobenzene and anisole could ionize PAAs through charge exchange processes taking place a direct interaction of the radical ion generated in the photoionization with the neutral molecules of analytes (Fig. 2C). Regarding the ionization efficiency in APPI, the highest ion abundances were observed using chlorobenzene as dopant (Fig. S4), although the signal intensity was slightly better with anisole for *p*-CA, *p*–C-*o*-MA, and BNZ and with toluene for *p*-AAB; 3,3′-DMB; and 4,4-M-2-CA. Therefore, as a compromise, chlorobenzene was selected as the most suitable dopant for the ionization of PAAs by APPI.

**Table 2:**
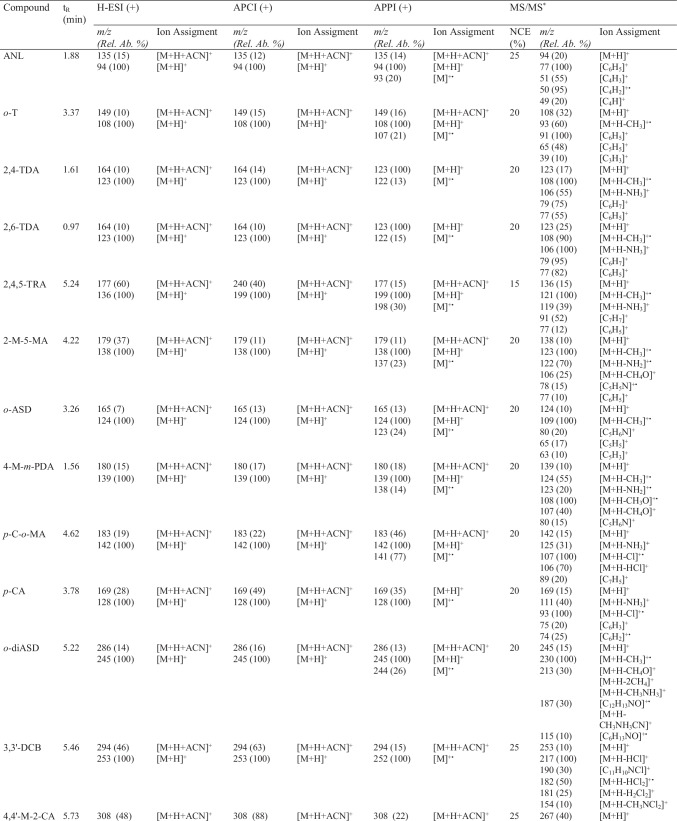

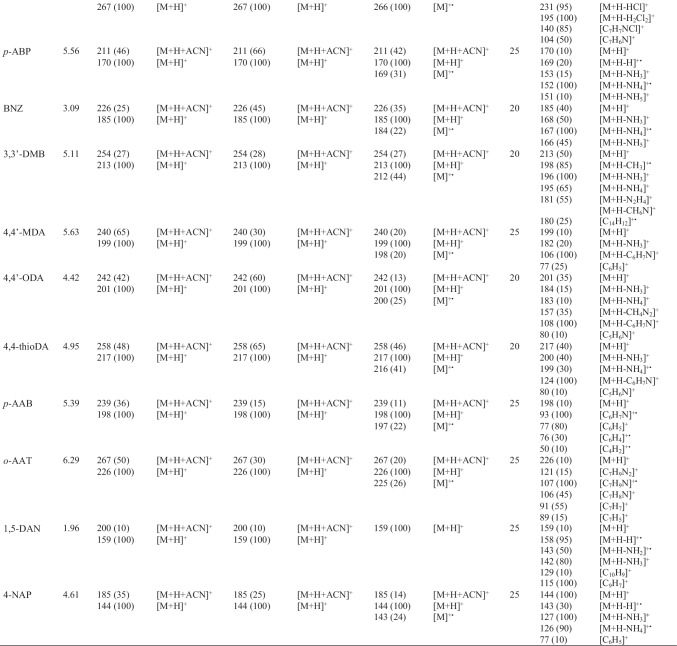
Retention time and assignment of ions and product ions observed in H-ESI, APCI, and APPI

^*^MS/MS studies were carried out using [M + H]^+^ as precursor ion in all cases

**Fig. 3:**
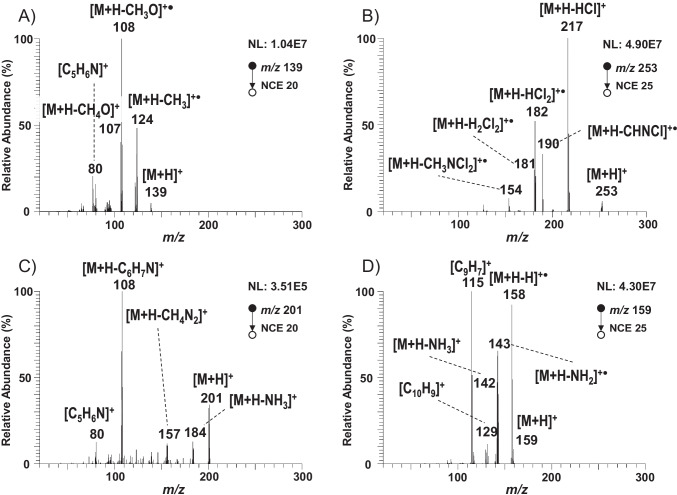
Product ion mass spectra of **A** 4-M-*m*-PDA; **B** 3,3′-DCL; **C** 4,4′-ODA; **D** 1,5-DAN using H-ESI in positive-ion mode

To improve the detection capability and selectivity of the method, as well as to guarantee the identification of PAAs, the fragmentation of [M + H]^+^ by tandem mass spectrometry was also studied. To the best of our knowledge, fragmentation studies and tandem mass spectral data to assign and characterize product ions have not been reported in the literature so far for all PAAs included in this study. Table 2 also shows the most relevant product ions observed in tandem mass spectrometry for each PAA. Generally, the fragmentation of the ion [M + H]^+^ showed a common pathway for most of the PAAs with one amino group. This fragmentation was due to the inductive cleavage of the C-N bond and the subsequent loss of the primary amino group, [M + H-NH_3_]^+^ or [M + H-NH_2_]^+^, whereas the diaminodiphenyl PAAs also yielded product ions due to the consecutive loss of the second amino group. Additionally, other characteristic aromatic product ions, such as *m/z* 77 [C_6_H_5_]^+^ and *m/z* 91 [C_7_H_7_]^+^, were also observed in the MS/MS spectra of some PAAs. ANL after losing the amino group is still fragmenting to yield product ions at *m/z* 51 [C_4_H_3_]^+^ and *m/z* 50 [C_4_H_2_]^+•^ as a consequence of the benzyl fragmentation. Besides, PAAs with a methyl group (*o*-T; 2,4-TDA; 2,6-TDA; and 2,4,5-TRA) also underwent the characteristic homolytic cleavage of the methyl group [M + H-CH_3_]^+•^ to yield product ions at *m/z* 93 for *o*-T; *m/z* 108 for 2,4-TDA and 2,6-TDA; and *m/z* 121 for 2,4,5-TRA. Regarding *ortho-*methyl ether PAAs (2-M-5-MA, *o*-ASD, 4-M-*m*-PDA, and *o*-diASD), they also lost a methyl group, due to the homolytic cleavage of the ether bond [M + H-CH_3_]^+•^ (Fig. 3A, m*/z* 124 for 4-M-*m*-PDA). Additionally, the homolytic bond cleavage in α to the oxygen induces the loss of the methoxy group, as it was also observed for 4-M-*m*-PDA (*m/z* 108, [M + H-CH_3_O]^+•^). However, both 2-M-5-MA and *o*-diASD showed the heterolytic cleavage of the C-O bond to yield the [M + H-CH_4_O]^+^ (*m/z* 107 and *m/z* 213, respectively). Regarding *para*- (*p*–C-*o*-MA and *p*-CA) and *ortho*- (3,3′-DCB and 4,4′-M-2-CA) chlorinated PAAs, the *para*-chlorinated PAAs showed a radical product ion due to the loss of chlorine [M + H-Cl]^+•^ (*m/z* 107 for *p*–C-*o*-MA and *m/z* 93 for *p*-CA), while *ortho*-chlorinated PAAs generated product ions due to the neutral loss of one or two molecules of hydrochloric acid, [M + H-HCl]^+^ (*m/z* 231) and [M + H-H_2_Cl_2_]^+^ (*m/z* 195) for 4,4′-M-2-CA, and [M + H-HCl]^+^ (*m/z* 217) and [M + H-HCl_2_]^+•^ (*m/z* 182) for 3,3′-DCB (Fig. 3B).

Regarding PAAs with two aromatic rings, those with a biphenyl structure (3,3′-DCB; *o*-diASD; *p*-ABP; BNZ; and 3,3′-DMB) mainly led to the generation of product ions through the characteristic fragmentation of the different functional groups (amino, methyl, and ether) previously described for other PAAs. Moreover, *o*-diASD and 3,3′-DCB also yielded product ions due to the loss of CHN and CN. For instance, the ion at *m/*z 187 in the MS/MS spectrum of *o*-diASD could be assigned to [M + H-C_2_H_6_N_2_]^+^, which might be generated as a consequence of the loss of CN from the isobaric product ion at *m/z* 213 [M + H-CH_6_N]^+^. Furthermore, the product ion at *m/z* 154 [M + H-CH_3_NCl_2_]^+^ for 3,3′-DCB could also be assigned to the consecutive loss of CHN from the product ion at *m/z* 181 [M + H-H_2_Cl_2_]^+^. On the other hand, the base peak for the dianilines (4,4′-MDA; 4,4′-ODA; and 4,4′-thioDA) was the product ion generated by the loss of aniline [M + H-C_6_H_7_N]^+^. However, these compounds also produced less abundant product ions due to the loss of the amine group, as can be observed in Fig. 3C for 4,4′-ODA (*m/z* 183 and *m/z* 184). Furthermore, the ion at *m/z* 80 for both 4,4′-ODA and 4,4′TDA could be assigned to the elimination of CO and CS from the ion [M + H-C_6_H_7_N]^+^. Finally, the ion at *m/z* 157 [M + H-CH_4_N_2_]^+^ observed for 4,4′-ODA could be assigned to the consecutive losses of NH_2_ and CO.

Regarding aminoazobenzene compounds (*p*-AAB and *o*-AAT), they undergo the homolytic cleavage of the C-N bond in α position to the azo group to generate the aromatic ions at *m/z* 77 [C_6_H_5_]^+^ and 93 [C_6_H_7_N]^+•^ for *p*-AAB and at *m/z* 107 [C_7_H_9_N]^+•^ and 91 [C_7_H_7_]^+^ for *o*-AAT. Additionally, *o*-AAT showed the breakage of the azo bond to yield two nitrile ions, the product ion at *m/z* 121 corresponding to the nitrile of the aminophenyl moiety [C_7_H_9_N_2_]^+^ and the ion at *m/z* 106 that could be assigned to the nitrile of the methylphenyl moiety [C_7_H_8_N]^+^. On the other hand, the two naphthyl compounds (1,5-DAN and 4-NAP) yielded the radical molecular ion due to the loss of one hydrogen [M + H–H]^+•^ (*m/z* 143 for 4-NAP and *m/z* 158 for 1,5-DAN) in addition to the typical loss of the amino group. However, at the MS/MS spectrum of 1,5-DAN the ion at *m/z* 129 [C_10_H_9_]^+^ is observed, which can be produced by the loss of the second amino group, as well as the ion at *m/z* 115 [CH_4_N_2_]^+^, probably due to the loss of CHN from the product ion at *m/z* 142 (Fig. 3D).

MS/MS fragmentation studies helped in the selection of the two most abundant and characteristic product ions required for quantitative and confirmatory purposes (Table S1) when applying the UHPLC-MS/MS method in multiple reaction monitoring (MRM) mode for the analysis of PAAs. The limits of detection (LODs) based on an S/N of 3 of the developed method using the three API sources were calculated to compare their performance and choose the most appropriate one for the determination of PAAs following the EU Regulation 1245/2020/EC [3]. LOD values were within the range of 0.4–2 μg kg^−1^ for the UHPLC-APCI-MS/MS method, whereas when using H-ESI and APPI sources, values were between 0.2–4 μg kg^−1^ and 0.7–8 μg kg^−1^, respectively.

Thereby, the LOD values of the developed UHPLC-APCI-MS/MS method were low enough to meet the Regulation 1245/2020/EC [3] for monitoring PAAs in migration test of food contact materials without the need to apply preconcentration steps prior the UHPLC-MS/MS analysis. Moreover, it should also be noted that APCI could be of particular relevance on the analysis of complex samples such as packaged food, since, as it is well-known that APCI shows lower matrix effects than ESI. Thereby, the proposed method would permit to a food control laboratory performing analysis of PAAs in both the migration test of FCM and the packaged food itself improving the laboratory throughput.

Table 3 summarized some quality parameters of the developed UHPLC-APCI-MS/MS method. Calibration curves, prepared in food simulant at concentration levels ranging from 1 to 500 μg kg^−1^ for most of PAAs (4,4′-MDA from 2 to 20,000 μg kg^−1^), showed good linearity with correlation coefficients (*r*) higher than 0.995 in all cases. Besides, both intra-day and inter-day precisions, expressed as relative standard deviation (RSD, %), were determined at three concentration levels, 2, 10, and 20 μg kg^−1^ and they were below 10% (*n* = 5) and 15% (*n* = 15, 3 days × 5 replicate analyses each), respectively. Moreover, trueness, calculated at the same concentration levels (*n* = 5) and expressed as the relative error (RE, %), was lower than 17% in all cases. Thereby, these results demonstrated the reliability of the developed UHPLC-APCI-MS/MS method for the quantification of the selected 23 PAAs at trace levels.

**Table 3:** Quality parameters of the developed UHPLC-APCI-MS/MS method

Compound	LOD	Intra-day precision (RSD, %)			Inter-day precision (RSD, %)			Trueness (RE, %)		
	(μg kg^−1^)	2 μg kg^−1^	10 μg kg^−1^	20 μg kg^−1^	2 μg kg^−1^	10 μg kg^−1^	20 μg kg^−1^	2 μg kg^−1^	10 μg kg^−1^	20 μg kg^−1^
ANL	0.04	10	5	1	10	3	2	15	7	6
*o*-T	0.6	7	2	2	10	1	2	11	8	4
2,4-TDA	2	−	1	5	−	11	5	−	− 17	− 8
2,6-TDA	2	−	7	4	−	1	6	−	− 12	− 2
2,4,5-TRA	1	5	2	1	5	3	2	8	3	1
2-M-5-MA	2	−	4	3	−	2	8	−	3	4
*o*-ASD	0.8	6	3	2	5	3	3	− 10	− 1	5
4-M-*m*-PDA	2	−	10	5	−	10	9	−	− 15	− 5
*p*–C-*o*-MA	0.3	8	5	3	15	8	3	16	7	1
*p*-CA	0.1	−	5	3	−	4	2	−	6	5
*o*-diASD	2	−	6	2	−	12	10	−	7	7
3,3′-DCB	1	−	7	5	−	6	4	−	8	4
4,4′-M-2-CA	1	10	4	2	12	6	5	13	3	3
*p*-ABP	2	−	4	4	−	6	5	−	7	4
BNZ	2	−	5	3	−	8	6	−	6	5
3,3′-DMB	1	−	6	5	−	2	1	−	1	9
4,4′-MDA	1	10	5	3	12	6	3	10	6	4
4,4′-ODA	2	10	6	4	15	9	5	16	7	6
4,4′-thioDA	2	–	5	4	–	11	4	–	0	5
*p*-AAB	0.1	−	7	6	−	5	4	−	10	1
*o*-AAT	2	12	8	6	14	9	5	12	5	4
1,5-DAN	2	−	6	2	−	9	5	−	− 4	11
2-NAP	0.6	8	4	3	13	2	3	1	1	1

### Sample analysis

**Fig. 4:**
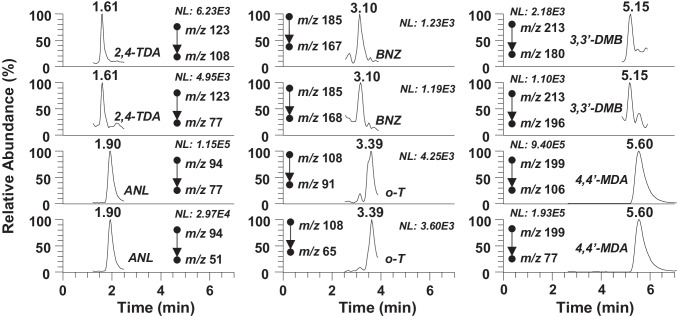
UHPLC-APCI-MS/MS chromatogram (quantitation and confirmation transitions) obtained from the analysis of PAAs in the 3rd migration test of sample 15

The developed UHPLC-APCI-MS/MS method was applied to evaluate the migration of PAAs from twenty black Nylon kitchenware samples. Table 4 summarizes sample details (number of samples, origin, and article type) in addition to the migration results of PAAs in the 3rd migration test (mean values and standard deviation of the concentration quantified in the food simulant B extracts), since the compliance shall be based on the concentration level determined in the third test [3]. In general, the concentration of PAAs that migrated into food simulant B decreased from the first to the third migration test, as expected (Table S2) [29]. Among samples analyzed, PAAs were in eighteen out of twenty at concentration above the legislated limit (2 μg kg^−1^). The 4,4′-MDA was the compound detected at the highest concentration (2900 to 18,519 μg kg^−1^), while ANL was detected in sixteen samples at concentration levels ranging from 2.5 to 108 μg kg^−1^. In the case of *o*-T, it was determined in seven samples at concentrations above the legislated migration limit (2.6–11.5 μg kg^−1^), whereas *o*-ASD was only detected in one sample at 82.0 μg kg^−1^. Additionally, *o*-T and _BNZ were identified in four and one samples, respectively, at concentrations below the legislated limit indicating that the analyzed materials complied with the legislation for these two PAAs. Regarding 2,6-TDA; 2,4-TDA; *p*-CA; 3,3′-DMB; and o-AZT, they were only detected in the 3rd migration test below the limit of quantitation, whereas *p*–C-*o*-MA was only determined in two samples in the 1st migration test, but never on the 3rd. As an example, Fig. 4 shows the extracted UHPLC-APCI-MS/MS chromatogram obtained from sample 15. These results indicate that the population can be exposed to PAAs through the use of these kitchen utensils, which might pose a risk to public health, and, hence, more efforts should be made to control and monitor PAAs in kitchenware plastic materials.

**Table 4:** PAA concentration (μg kg^−1^) obtained in the 3rd migration tests (food simulant B) performed with black kitchenware samples by UHPLC-APCI-MSMS

Sample type (Manufacture country)		Migration test	Compound concentration (standard deviation)										
			ANL	*o*-T	2,4-TDA	2,6-TDA	*o*-ASD	*p*–C-*o*-MA	*p*-CA	BNZ	3,3′-DMB	4,4′-MDA	*o*-AAT
1	Spatula (China)	3rd	81 (13)	n.d	n.d	n.d	n.d	n.d	n.d	n.d	n.d	n.d	n.d
2	Spatula (China)	3rd	4.2 (0.4)	1.8 (0.3)	n.d	n.d	n.d	n.d	< LOQ	n.d	n.d	n.d	n.d
3	Slotted spatula (China)	3rd	16 (1)	n.d	n.d	n.d	n.d	n.d	n.d	n.d	n.d	n.d	n.d
4	Slotted spatula (China)	3rd	23 (3)	n.d	n.d	n.d	n.d	n.d	< LOQ	n.d	n.d	n.d	n.d
5	Slotted spatula (China)	3rd	2.5 (0.3)	< LOQ	n.d	n.d	n.d	n.d	n.d	n.d	n.d	12,593 (1153)	n.d
6	Spoon (China)	3rd	122 (9)	n.d	< LOQ	n.d	< LOQ	n.d	n.d	n.d	n.d	18,519 (893)	n.d
7	Spoon (China)	3rd	18 (2)	n.d	n.d	n.d	n.d	n.d	n.d	n.d	n.d	14,380 (1728)	n.d
8	Spoon (Not declared)	3rd	< LOQ	6.2 (0.4)	n.d	n.d	82 (1)	n.d	n.d	n.d	n.d	n.d	n.d
9	Spoon (Spain)	3rd	82 (8)	3.7 (0.6)	n.d	n.d	n.d	n.d	< LOQ	n.d	n.d	13,373 (1217)	< LOQ
10	Spoon (China)	3rd	47 (10)	1.6 (0.4)	n.d	n.d	n.d	n.d	< LOQ	< LOQ	n.d	8790 (991)	< LOQ
11	Spoon (China)	3rd	108 (11)	3.2 (0.3)	n.d	n.d	n.d	n.d	n.d	< LOQ	n.d	n.d	n.d
12	Slotted spoon (China)	3rd	96 (10)	4.7 (0.6)	< LOQ	n.d	n.d	n.d	n.d	< LOQ	n.d	n.d	< LOQ
13	Slotted spoon (China)	3rd	37 (2)	1.4 (0.1)	n.d	n.d	n.d	n.d	n.d	< LOQ	n.d	10,811 (671)	n.d
14	Slotted spoon (China)	3rd	n.d	n.d	n.d	n.d	n.d	n.d	n.d	n.d	n.d	n.d	n.d
15	Slotted spoon (China)	3rd	33 (6)	2.6 (0.2)	< LOQ	n.d	n.d	n.d	n.d	1.8 (0.2)	< LOQ	2920 (77)	n.d
16	Slotted turner (Spain)	3rd	62 (3)	1.7 (0.1)	n.d	n.d	n.d	n.d	< LOQ	n.d	n.d	15,621 (718)	< LOQ
17	Slotted turner (China)	3rd	2.8 (0.4)	< LOQ	n.d	n.d	n.d	n.d	< LOQ	n.d	n.d	n.d	n.d
18	Slotted turner (China)	3rd	31.1 (0.4)	6.9 (0.2)	< LOQ	< LOQ	n.d	n.d	< LOQ	n.d	n.d	13,851 (472)	n.d
19	Potato masher (China)	3rd	n.d	< LOQ	n.d	n.d	n.d	n.d	< LOQ	n.d	n.d	n.d	n.d
20	Spaghetti server (Spain)	3rd	n.d	11.5 (0.4)	n.d	n.d	n.d	n.d	n.d	n.d	n.d	n.d	n.d

*n.d.*, no detected (< LOD)

## Conclusions

This work has demonstrated, for the first time, the good chromatographic performance of an LC column with a pentafluorophenylpropyl (PFPP) stationary phase for the simultaneous separation of 23 PAAs in less than 6.5 min and using an acidic mobile phase free of ion-pair reagents, which enhanced the ionization of PAAs in APCI-MS. Although the protonated molecule [M + H]^+^ predominated in the API mass spectrum of the PAAs, these compounds showed a tendency to generate acetonitrile adduct ions [M + H + ACN]^+^ in the three API sources. However, the thermal assistance (vaporizer temperature up to 400 °C) minimized the presence of these adducts. Tandem mass spectrometry studies allowed the identification of the most characteristic product ions of 23 PAAs and showed, for the first time, the MS/MS fragmentation behavior of this family of compounds. Among the atmospheric pressure ionization sources evaluated (H-ESI, APCI, and APPI), the best ionization efficiency of PAAs was observed when using APCI in positive-ion mode. The UHPLC-APCI-MS/MS (MRM mode) method achieved LOD values low enough to meet the EU-specific migration limits of PAAs in food contact materials (2 μg kg^−1^). Quality parameters demonstrated the good performance of the developed method and its feasibility for the determination of PAAs in the migration test performed to black Nylon kitchenware samples. Eighteen out of twenty samples showed the presence of PAAs at concentration levels above the legislation limit on the 3rd migration test. PAAs detected at the highest concentration level were 4,4′-MDA (2900–18,519 μg kg^−1^) and ANL (16–108 μg kg^−1^). These results demonstrate that the developed method can be proposed for the simultaneous determination 23 PAAs in migration tests of black Nylon kitchenware.

## Supplementary Information

Below is the link to the electronic supplementary material.

Supplementary file1 (PDF 1189 KB)
