# Supplementary material for: Ultra-high-performance liquid chromatography-atmospheric pressure ionization-tandem mass spectrometry method for the migration studies of primary aromatic amines from food contact materials
Source: Anal Bioanal Chem. 2022 Mar 2;414(9):3137–51. doi: 10.1007/s00216-022-03946-3 (PMC8934768; doi:10.1007/s00216-022-03946-3)
Supplement: Supplementary file 1 — Supplementary file1 (PDF 1189 KB) [file 216_2022_3946_MOESM1_ESM.pdf]

Supplementary material

# Ultra-high-performance liquid chromatography-atmospheric pressure ionization-tandem mass spectrometry method for the migration studies of primary aromatic amines from food contact materials

A. Arrizabalaga-Larrañaga[(1)]*, P. de Juan-de Juan[(1)], C. Bressan[(1)], M. Vázquez-Espinosa[(2)], A. V. González-de-Peredo[(2)], F. J. Santos[(1)], E. Moyano[(1)]

[(1)] Department of Chemical Engineering and Analytical Chemistry, University of Barcelona. Av. Diagonal 645, E-08028 Barcelona, Spain

[(2)] Department of Analytical Chemistry, Faculty of Sciences, University of Cadiz, Agrifood Campus of International Excellence (ceiA3), IVAGRO, 11510 Puerto Real, Cadiz, Spain

* Corresponding author: Ane Arrizabalaga-Larrañaga

Department of Chemical Engineering and Analytical Chemistry University of Barcelona
Av. Diagonal 645, E-08028, Barcelona, Spain
E-mail: a.arrizabalaga@ub.edu

## Table of Contents

**Supplementary Tables** ........ 2

**Table S1:** MRM transitions, optimum collision energies (NCEs) and ion ratios used in the UHPLC–APCI–MS/MS method. ........ 2

**Table S2.** PAA concentration (μg $kg^{-1}$) obtained in the migration tests (simulant B) performed with black kitchenware samples by UHPLC–APCI–MSMS. ........ 3

**Supplementary Figures** ........ 6

**Figure S1:** UHPLC−ESI−MS chromatograms obtained from a solution of PAAs injected in (A) C18 column (methanol:water, *v/v*), (B) phenyl-hexyl column (methanol:water (0.1% formic acid, *v/v*)), and (C) HILIC column (acetonitrile:0.1% aqueous formic acid (90:10, *v/v*)). ........ 6

**Figure S2:** The plot of retention time of PAAs *vs.* the percentage of organic modifier. The standard mixture of 23 PAAs was injected in isocratic elution mode using different percentage of organic modifier (10-90%) in mobile phases based on mixtures of methanol:water (0.1% formic acid) and acetonitrile:water (0.1% formic acid) ........ 7

**Figure S3:** Effect of vaporizer temperature on the response of 2-M-5-MA in APCI source at (A) 300ºC and (B) 400ºC . ........ 8

**Figure S4:** Effect of different APPI-dopants (post-column addition of 5% v/v) on the response of the studied PAAs using UHPLC–APPI–MS. ........ 9

# Supplementary Tables

**Table S1:** MRM transitions, optimum collision energies (NCEs) and ion ratios used in the UHPLC–APCI–MS/MS method.

| Compound | Precursor ion (*m/z*) | Quantitation | | Confirmation | | Ion Ratio (RSD %)[a] |
|---|---|---|---|---|---|---|
| | | Product ion (*m/z*) | NCE (V) | Product ion (*m/z*) | NCE (V) | |
| ANL | 94 | 77 | 18 | 51 | 32 | 4.0 (3) |
| *o*-T | 108 | 91 | 17 | 65 | 27 | 2.6 (3) |
| 2,4-TDA | 123 | 108 | 15 | 77 | 30 | 1.4 (4) |
| 2,6-TDA | 123 | 106 | 15 | 77 | 28 | 1.4 (1) |
| 2,4,5-TRA | 136 | 121 | 15 | 91 | 23 | 1.4 (1) |
| 2-M-5-MA | 138 | 123 | 20 | 122 | 25 | 3.5 (2) |
| *o*-ASD | 124 | 109 | 15 | 80 | 30 | 3.8 (2) |
| 4-M-*m*-PDA | 139 | 108 | 15 | 124 | 15 | 1.3 (5) |
| *p*-C-*o*-MA | 142 | 107 | 15 | 106 | 25 | 1.8 (4) |
| *p*-CA | 128 | 93 | 17 | 75 | 30 | 3.4 (5) |
| *o*-diASD | 245 | 187 | 35 | 230 | 20 | 1.2 (1) |
| 3,3'-DCB | 253 | 217 | 20 | 182 | 27 | 1.4 (8) |
| 4,4’-M-2-CA | 267 | 231 | 20 | 195 | 28 | 1.2 (8) |
| *p*-ABP | 170 | 152 | 30 | 153 | 20 | 2.7 (3) |
| BNZ | 185 | 167 | 27 | 168 | 17 | 1.1 (2) |
| 3,3’-DMB | 213 | 180 | 33 | 196 | 20 | 2.3 (4) |
| 4,4’-MDA | 199 | 106 | 25 | 77 | 44 | 4.4 (2) |
| 4,4’-ODA | 201 | 108 | 20 | 80 | 30 | 2.1 (5) |
| 4,4’-thioDA | 217 | 124 | 20 | 200 | 17 | 3.0 (8) |
| *p*-AAB | 198 | 77 | 20 | 93 | 20 | 2.7 (3) |
| *o*-AAT | 226 | 107 | 25 | 89 | 45 | 1.3 (1) |
| 1,5-DAN | 159 | 115 | 30 | 143 | 20 | 1.5 (6) |
| 4-NAP | 144 | 127 | 23 | 77 | 35 | 2.6 (4) |

[a] RSD,% (n=5)

**Table S2.** PAA concentration (μg kg$^{-1}$) obtained in the migration tests (simulant B) performed with black kitchenware samples by UHPLC–APCI–MSMS.

| Sample type (Manufacture country) | | Migration test | Compound concentration (standard deviation) | | | | | | | | | | |
|---|---|---|---|---|---|---|---|---|---|---|---|---|---|
| | | | ANL | *o*-T | 2,4-TDA | 2,6-TDA | *o*-ASD | *p*-C-*o*-MA | *p*-CA | BNZ | 3,3'-DMB | 4,4'-MDA | *o*-AAT |
| 1 | Spatula | 3rd | 81 (13) | n.d | n.d | n.d | n.d | n.d | n.d | n.d | n.d | n.d | n.d |
| | (China) | 2nd | 97 (9) | n.d | n.d | n.d | n.d | n.d | n.d | n.d | n.d | n.d | n.d |
| | | 1st | 127 (5) | n.d | n.d | n.d | n.d | n.d | n.d | n.d | n.d | n.d | n.d |
| 2 | Spatula | 3rd | 4.2 (0.4) | 1.8 (0.3) | n.d | n.d | n.d | n.d | < LOQ | n.d | n.d | n.d | n.d |
| | (China) | 2nd | 5.1 (0.5) | 2.0 (0.1) | n.d | n.d | n.d | n.d | < LOQ | n.d | n.d | n.d | n.d |
| | | 1st | 7 (1) | 2.2 (0.3) | n.d | n.d | n.d | n.d | < LOQ | n.d | n.d | n.d | n.d |
| 3 | Slotted spatula | 3rd | 16 (1) | n.d | n.d | n.d | n.d | n.d | n.d | n.d | n.d | n.d | n.d |
| | (China) | 2nd | 22 (1) | n.d | n.d | n.d | n.d | n.d | n.d | n.d | n.d | n.d | n.d |
| | | 1st | 57 (2) | n.d | n.d | n.d | n.d | n.d | n.d | n.d | n.d | n.d | n.d |
| 4 | Slotted spatula | 3rd | 23 (3) | n.d | n.d | n.d | n.d | n.d | < LOQ | n.d | n.d | n.d | n.d |
| | (China) | 2nd | 31 (4) | n.d | n.d | n.d | n.d | n.d | < LOQ | n.d | n.d | n.d | n.d |
| | | 1st | 74 (6) | n.d | n.d | n.d | < LOQ | 3.1 (0.1) | < LOQ | n.d | n.d | n.d | n.d |
| 5 | Slotted spatula | 3rd | 2.5 (0.3) | < LOQ | n.d | n.d | n.d | n.d | n.d | n.d | n.d | 12593 (1,153) | n.d |
| | (China) | 2nd | 3.4 (0.4) | 1.6 (0.1) | n.d | n.d | n.d | n.d | n.d | n.d | n.d | 18722 (1,557) | n.d |
| | | 1st | 9.44 (0.09) | 3.4 (0.2) | n.d | n.d | n.d | n.d | n.d | n.d | n.d | 56582 (2,283) | n.d |
| 6 | Spoon | 3rd | 122 (9) | n.d | < LOQ | n.d | < LOQ | n.d | n.d | n.d | n.d | 18519 (893) | n.d |
| | (China) | 2nd | 159 (6) | n.d | < LOQ | n.d | < LOQ | n.d | n.d | n.d | n.d | 24632 (933) | n.d |
| | | 1st | 393 (34) | n.d | < LOQ | n.d | 3.16 (0.07) | 3.5 (0.3) | n.d | n.d | n.d | 75842 (5,409) | n.d |
| 7 | Spoon | 3rd | 18 (2) | n.d | n.d | n.d | n.d | n.d | n.d | n.d | n.d | 14380 (1,728) | n.d |
| | (China) | 2nd | 31 (2) | n.d | n.d | n.d | n.d | n.d | n.d | n.d | n.d | 22764 (4,481) | n.d |
| | | 1st | 81 (5) | n.d | n.d | n.d | n.d | n.d | 6.3 (0.3) | n.d | n.d | 59184 (3,965) | n.d |
| 8 | Spoon | 3rd | < LOQ | 6.2 (0.4) | n.d | n.d | 82 (1) | n.d | n.d | n.d | n.d | n.d | n.d |
| | (Not declared) | 2nd | < LOQ | 7.4 (0.4) | n.d | n.d | 111 (3) | n.d | n.d | n.d | n.d | n.d | n.d |
| | | 1st | 13.7 (0.9) | 10.0 (0.2) | n.d | < LOQ | 160 (7) | n.d | n.d | n.d | n.d | n.d | n.d |

n.d.: no detected (<LOD).

**Table S2 (cont.).** PAA concentration (μg kg$^{-1}$) obtained in the migration tests (simulant B) performed with black kitchenware samples by UHPLC–APCI–MSMS.

| Sample type (Manufacture country) | | Migration test | Compound concentration (standard deviation) ANL | *o*-T | 2,4-TDA | 2,6-TDA | *o*-ASD | *p*-C-*o*-MA | *p*-CA | BNZ | 3,3'-DMB | 4,4'-MDA | *o*-AAT |
|---|---|---|---|---|---|---|---|---|---|---|---|---|---|
| 9 | Spoon | 3rd | 82 (8) | 3.7 (0.6) | n.d | n.d | n.d | n.d | < LOQ | n.d | n.d | 13373 (1,217) | < LOQ |
| | (Spain) | 2nd | 101 (4) | 4.7 (0.9) | n.d | n.d | n.d | n.d | < LOQ | n.d | n.d | 18250 (1,507) | < LOQ |
| | | 1st | 181 (5) | 10.9 (0.4) | n.d | n.d | n.d | n.d | 10.6 (0.5) | < LOQ | n.d | 53030 (1,358) | 2.5 (0.1) |
| 10 | Spoon | 3rd | 47 (10) | 1.6 (0.4) | n.d | n.d | n.d | n.d | < LOQ | < LOQ | n.d | 8790 (991) | < LOQ |
| | (China) | 2nd | 56 (10) | 2.2 (0.8) | n.d | n.d | n.d | n.d | < LOQ | < LOQ | n.d | 11923 (563) | < LOQ |
| | | 1st | 113 (15) | 6 (2) | n.d | n.d | n.d | n.d | 8 (1) | 5 (1) | n.d | 40976 (7,213) | 1.6 (0.7) |
| 11 | Spoon | 3rd | 108 (11) | 3.2 (0.3) | n.d | n.d | n.d | n.d | n.d | < LOQ | n.d | n.d | n.d |
| | (China) | 2nd | 157 (7) | 4.8 (0.2) | n.d | n.d | n.d | n.d | n.d | < LOQ | n.d | n.d | n.d |
| | | 1st | 340 (36) | 12.8 (0.8) | n.d | n.d | n.d | n.d | n.d | 9.14 (0.07) | n.d | n.d | n.d |
| 12 | Slotted spoon | 3rd | 96 (10) | 4.7 (0.6) | < LOQ | n.d | n.d | n.d | n.d | < LOQ | n.d | n.d | < LOQ |
| | (China) | 2nd | 123 (15) | 5.8 (0.9) | < LOQ | n.d | n.d | n.d | n.d | < LOQ | n.d | n.d | < LOQ |
| | | 1st | 237 (18) | 16 (1) | < LOQ | n.d | n.d | n.d | n.d | 6.5 (0.6) | n.d | n.d | 1.3 (0.3) |
| 13 | Slotted spoon | 3rd | 37 (2) | 1.4 (0.1) | n.d | n.d | n.d | n.d | n.d | < LOQ | n.d | 10811 (671) | n.d |
| | (China) | 2nd | 56 (3) | 2.4 (0.4) | n.d | n.d | n.d | n.d | n.d | < LOQ | n.d | 15112 (1,106) | n.d |
| | | 1st | 99 (11) | 5.3 (0.1) | n.d | n.d | n.d | n.d | < LOQ | < LOQ | n.d | 35125 (1,347) | 1.4 (0.5) |
| 14 | Slotted spoon | 3rd | n.d | n.d | n.d | n.d | n.d | n.d | n.d | n.d | n.d | n.d | n.d |
| | (China) | 2nd | n.d | n.d | n.d | n.d | n.d | n.d | n.d | n.d | n.d | n.d | n.d |
| | | 1st | n.d | n.d | n.d | n.d | n.d | n.d | n.d | n.d | n.d | n.d | n.d |
| 15 | Slotted spoon | 3rd | 33 (6) | 2.6 (0.2) | < LOQ | n.d | n.d | n.d | n.d | 1.8 (0.2) | < LOQ | 2920 (77) | n.d |
| | (China) | 2nd | 45 (15) | 3.7 (0.3) | < LOQ | n.d | n.d | n.d | n.d | 2.3 (0.1) | < LOQ | 4102 (214) | n.d |
| | | 1st | 72 (11) | 7.5 (0.3) | 20 (2) | n.d | n.d | n.d | n.d | 4.8 (0.3) | < LOQ | 10108 (363) | n.d |
| 16 | Slotted turner | 3rd | 62 (3) | 1.7 (0.1) | n.d | n.d | n.d | n.d | < LOQ | n.d | n.d | 15621 (718) | < LOQ |
| | (Spain) | 2nd | 81 (4) | 2.2 (0.4) | n.d | n.d | n.d | n.d | < LOQ | n.d | n.d | 20348 (1,880) | < LOQ |
| | | 1st | 174 (10) | 5.4 (0.2) | n.d | n.d | n.d | n.d | 11 (1) | n.d | n.d | 55201 (4,195) | 3.0 (0.4) |

n.d.: no detected (<LOD).

**Table S2 (cont.).** PAA concentration (µg kg$^{-1}$) obtained in the migration tests (simulant B) performed with black kitchenware samples by UHPLC–APCI–MSMS.

| Sample type (Manufacture country) | | Migration test | Compound concentration (standard deviation) | | | | | | | | | | |
|---|---|---|---|---|---|---|---|---|---|---|---|---|---|
| | | | ANL | *o*-T | 2,4-TDA | 2,6-TDA | *o*-ASD | *p*-C-*o*-MA | *p*-CA | BNZ | 3,3'-DMB | 4,4'-MDA | *o*-AAT |
| 17 | Slotted turner | 3rd | 2.8 (0.4) | < LOQ | n.d | n.d | n.d | n.d | < LOQ | n.d | n.d | n.d | n.d |
| | (China) | 2nd | 3.9 (0.4) | 1.6 (0.3) | n.d | n.d | n.d | n.d | < LOQ | n.d | n.d | n.d | n.d |
| | | 1st | 11.3 (0.7) | 3.6 (0.4) | n.d | n.d | n.d | n.d | < LOQ | n.d | n.d | n.d | n.d |
| 18 | Slotted turner | 3rd | 31.1 (0.4) | 6.9 (0.2) | < LOQ | < LOQ | n.d | n.d | < LOQ | n.d | n.d | 13851 (472) | n.d |
| | (China) | 2nd | 40.1 (0.9) | 8.8 (0.3) | < LOQ | < LOQ | n.d | n.d | < LOQ | n.d | n.d | 18131 (511) | n.d |
| | | 1st | 91 (2) | 19 (2) | 18 (2) | < LOQ | < LOQ | n.d | 5.5 (0.6) | < LOQ | n.d | 54567 (988) | < LOQ |
| 19 | Potato masher | 3rd | n.d | < LOQ | n.d | n.d | n.d | n.d | < LOQ | n.d | n.d | n.d | n.d |
| | (China) | 2nd | n.d | < LOQ | n.d | n.d | n.d | n.d | < LOQ | n.d | n.d | n.d | n.d |
| | | 1st | n.d | 2.0 (0.2) | n.d | n.d | n.d | n.d | < LOQ | n.d | n.d | n.d | n.d |
| 20 | Spaghetti server | 3rd | n.d | 11.5 (0.4) | n.d | n.d | n.d | n.d | n.d | n.d | n.d | n.d | n.d |
| | (Spain) | 2nd | n.d | 13 (1) | n.d | n.d | n.d | n.d | n.d | n.d | n.d | n.d | n.d |
| | | 1st | n.d | 17 (2) | n.d | n.d | n.d | n.d | n.d | n.d | n.d | n.d | n.d |

n.d.: no detected (<LOD).

# Supplementary Figures

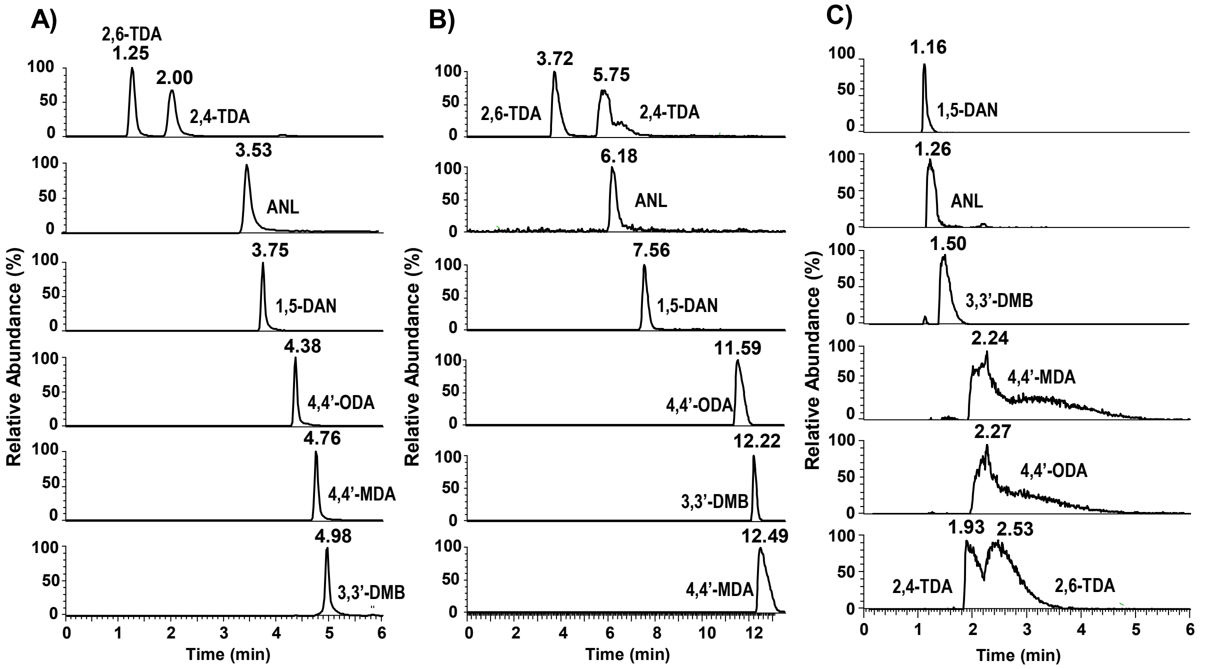


**Figure S1:** UHPLC−ESI−MS chromatograms obtained from a solution of PAAs injected in (A) C18 column (methanol:water, *v/v*), (B) phenyl-hexyl column (methanol:water (0.1% formic acid, *v/v*)), and (C) HILIC column (acetonitrile:0.1% aqueous formic acid (90:10, *v/v*)).

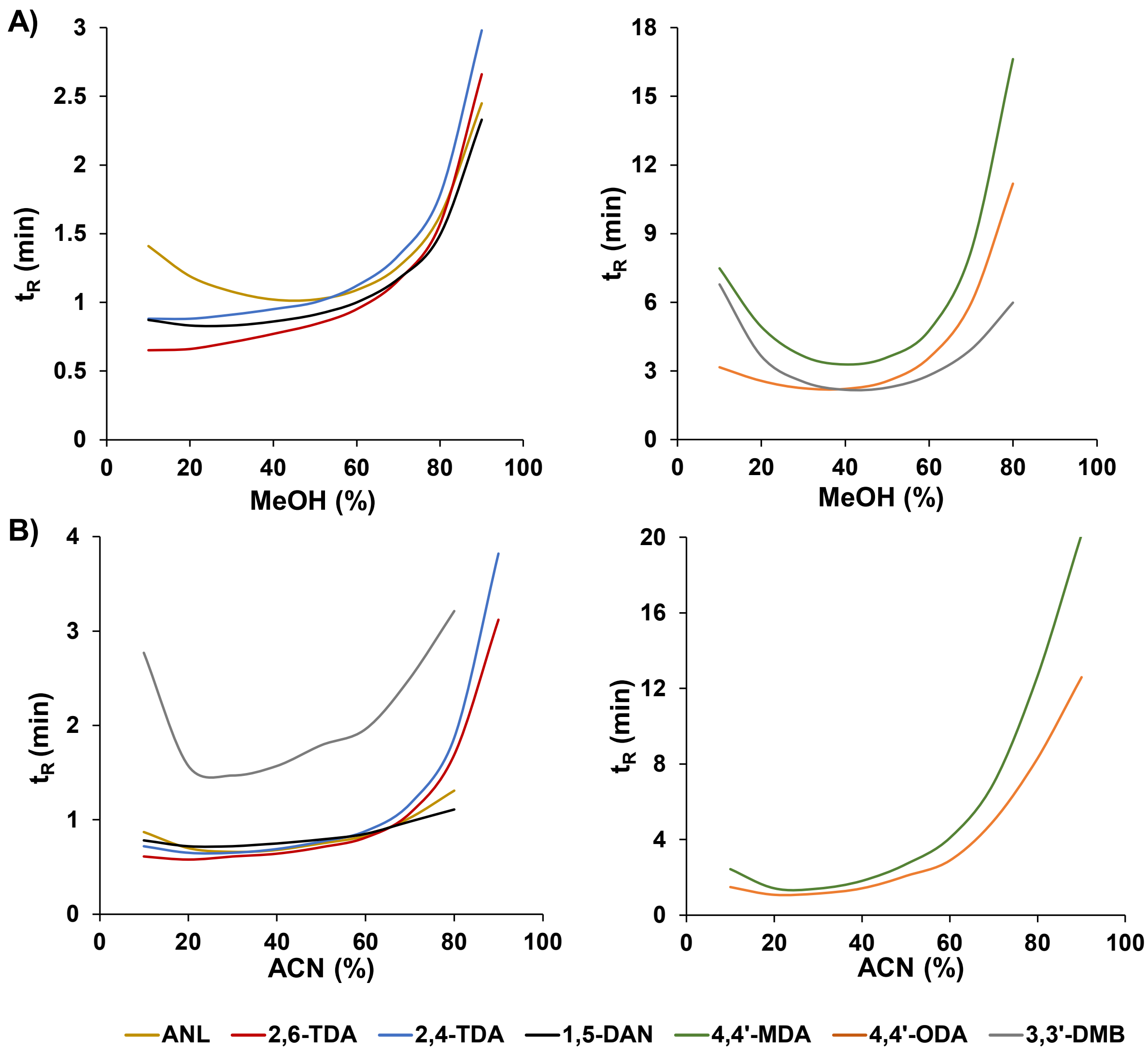


**Figure S2:** The plot of retention time of PAAs *vs.* the percentage of organic modifier. The standard mixture of 23 PAAs was injected in isocratic elution mode using different percentage of organic modifier (10-90%) in mobile phases based on mixtures of methanol:water (0.1% formic acid) and acetonitrile:water (0.1% formic acid)

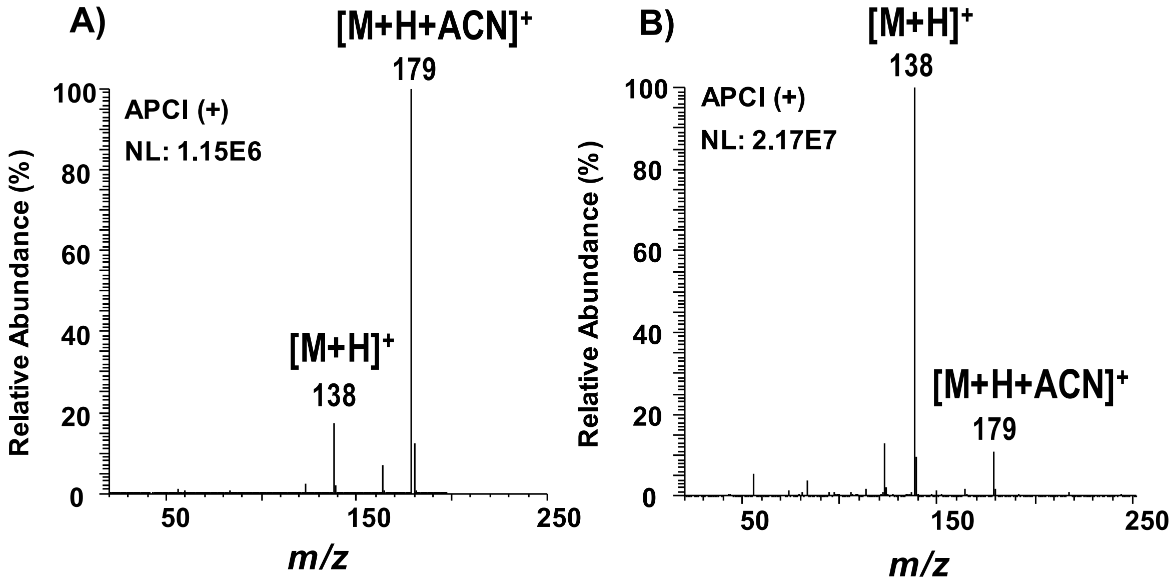


**Figure S3:** Effect of vaporizer temperature on the response of 2-M-5-MA in APCI source at (A) 300ºC and (B) 400ºC .

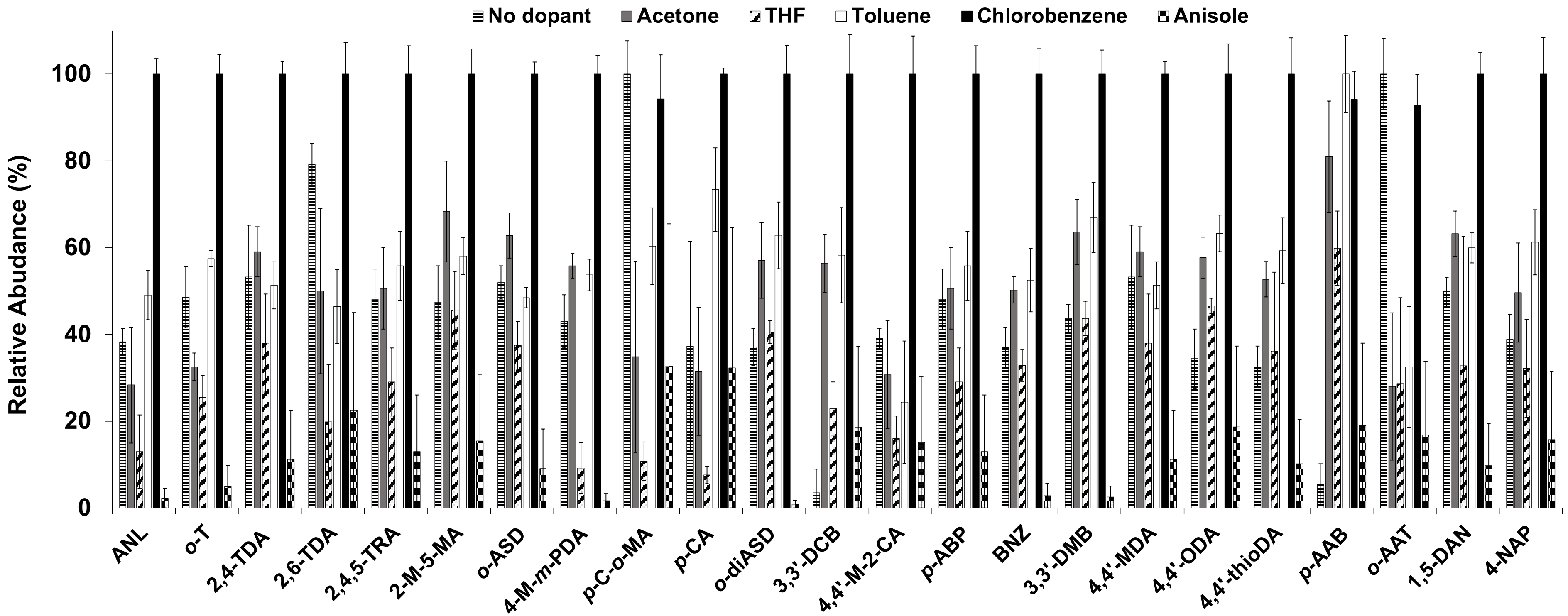


**Figure S4:** Effect of different APPI-dopants (post-column addition of 5% *v/v*) on the response of the studied PAAs using UHPLC–APPI–MS.
